# Promise and Progress of an HIV-1 Cure by Adeno-Associated Virus Vector Delivery of Anti-HIV-1 Biologics

**DOI:** 10.3389/fcimb.2020.00176

**Published:** 2020-04-23

**Authors:** Matthew R. Gardner

**Affiliations:** Department of Immunology and Microbiology, The Scripps Research Institute, Jupiter, FL, United States

**Keywords:** adeno-associate virus, HIV-1, HIV-1 cure, broadly neutralizing antibodies (bNAbs), eCD4-Ig

## Abstract

Despite the success of antiretroviral therapy (ART) at suppressing HIV-1 infection, a cure that eradicates all HIV-1-infected cells has been elusive. The latent viral reservoir remains intact in tissue compartments that are not readily targeted by the host immune response that could accelerate the rate of reservoir decline during ART. However, over the past decade, numerous broadly neutralizing antibodies (bNAbs) have been discovered and characterized. These bNAbs have also given rise to engineered antibody-like inhibitors that are just as or more potent than bNAbs themselves. The question remains whether bNAbs and HIV-1 inhibitors will be the effective “kill” to a shock-and-kill approach to eliminate the viral reservoir. Additional research over the past few years has sought to develop recombinant adeno-associated virus (rAAV) vectors to circumvent the need for continual administration of bNAbs and maintain persistent expression in a host. This review discusses the advancements made in using rAAV vectors for the delivery of HIV-1 bNAbs and inhibitors and the future of this technology in HIV-1 cure research. Numerous groups have demonstrated with great efficacy that rAAV vectors can successfully express protective concentrations of bNAbs and HIV-1 inhibitors. Yet, therapeutic concentrations, especially in non-human primate (NHP) models, are not routinely achieved. As new studies have been reported, more challenges have been identified for utilizing rAAV vectors, specifically how the host immune response limits the attainable concentrations of bNAbs and inhibitors. The next few years should provide improvements to rAAV vector delivery that will ultimately show whether they can be used for expressing bNAbs and HIV-1 inhibitors to eliminate the HIV-1 viral reservoir.

## Introduction

The 2019 UNAIDS Report and World Health Organization both estimate that there are ~38 million people living with HIV-1 infection (UNAIDS, 2019; WHO, 2019). Of those individuals, almost 25 million have access to antiretroviral therapy (ART) (UNAIDS, 2019). Various studies have shown the half-life of the HIV-1 reservoir can range somewhere between 44 months and 13 years and in some cohorts, no decay was observed at all (Siliciano et al., 2003; Chun et al., 2007; Besson et al., 2014; Bachmann et al., 2019). Thus, lifelong ART is required to maintain viral suppression and achieve the best health outcomes in the majority of individuals.

As more individuals living with HIV-1 receive daily ART, there is a push to investigate new means to cure the disease. Two definitions are routinely used in the field (Deeks et al., 2016). The first definition is a traditional cure, where the virus is eradicated or eliminated from the individual, including the latent viral reservoir. The second definition is a “functional cure” or remission, where an individual has their viremia suppressed to levels that limit transmission, and the individual does not progress to AIDS. Current ART falls in the second category, and thus, new therapies are needed in order to fully cure the disease.

Broadly neutralizing antibodies (bNAbs) and engineered HIV-1 inhibitors could serve as the new drugs that could help eliminate the latent reservoir and cure individuals with HIV-1. However, current uses of bNAbs in therapy require continual bNAb infusions along with reactivation of the latent viral reservoir, which could be considered a costly endeavor that is not practical for areas in developing nations with larger numbers of individuals living with HIV-1. Yet, bNAbs and antibody-like inhibitors utilize the Fc region that promotes effector functions via antibody interactions with immune cells (Lu et al., 2018), properties that may be useful for an HIV-1 cure. These effector functions include antibody-dependent complement-mediated lysis (ADCML) (Mujib et al., 2017), antibody-dependent cell-mediated cytotoxicity (ADCC) (Bruel et al., 2016; Von Bredow et al., 2016), and antibody-dependent cellular phagocytosis (ADCP) (Julg et al., 2017; Mayer et al., 2017). Through these mechanisms, bNAbs can identify and kill HIV-1-infected cells within the latent viral reservoir.

This review discusses the use of recombinant adeno-associated virus (rAAV) vectors to overcome the challenges associated with passive infusion of bNAbs and other HIV-1 inhibitors. The past decade has yielded numerous studies that evaluate the delivery of these biologics in different models as well as their protective and therapeutic efficacy. However, as more studies are reported, new challenges are identified that will need to be resolved for rAAV vectors to be employed in the clinic. Additionally, questions still remain as to whether bNAbs and other HIV-1 inhibitors can increase the rate of decay of the viral reservoir and provide the necessary means to cure individuals living with HIV-1. Despite these hurdles, rAAV delivery of bNAbs and HIV-1 inhibitors is a promising approach that warrants further investigation.

## Broadly Neutralizing Antibodies That Target the HIV-1 Envelope Glycoprotein

Due to the limitations of current antiretroviral therapies for reducing the viral reservoir and eradicating infected cells while viral loads are suppressed, there is a clear need for new drugs to treat those living with HIV-1. Antibodies are an obvious alternative due to their ability to not only bind and neutralize pathogens but because of their effector functions mediated by the Fc region. Prior to 2009, small molecule inhibitors were the only viable pathway for HIV-1 therapy because biologics, specifically antibodies, were not broad or potent enough to be used as therapeutics. Advances in single B cell sorting along with identification of HIV-1+ individuals with neutralizing sera from large patient cohorts allowed Walker et al. to usher in the second generation of bNAbs with their discovery of PG9 and PG16 (Walker et al., 2009). Since then, numerous bNAbs have been identified and characterized that potently neutralize hundreds of HIV-1 primary isolates in TZM-bl neutralization assays. These bNAbs are categorized based on the epitope of the HIV-1 envelope glycoprotein (Env) that they target. PG9 and PG16 are members of the class of bNAbs that target the V2 apex of Env. This class now includes PGT145 (Walker et al., 2011), PGDM1400 (Sok et al., 2014), and CAP256-VRC26.25 (Doria-Rose et al., 2016). One class that demonstrates extreme breadth is the CD4-binding site antibodies which includes VRC01 (Wu et al., 2010), VRC07-523LS (Rudicell et al., 2014), 3BNC117 (Scheid et al., 2011), N6 (Huang et al., 2016a), N49P7 (Sajadi et al., 2018), and 1-18 (Schommers et al., 2020). 10-1074 (Mouquet et al., 2012), PGT121, PGT122, and PGT128 (Walker et al., 2011) are examples bNAbs that target the N332 glycan near the base of V3 loop (V3g) of Env. 10E8 is a bNAb that targets the membrane proximal external region (MPER) of gp41 on Env (Huang et al., 2012) and has since been engineered for enhanced biophysical properties like improved solubility (10E8v4) (Kwon et al., 2016). More recent research identified newer epitopes on Env previously not known to be targets of antibody responses. One of these epitopes included the gp120/gp41 interface with examples including PGT151 (Falkowska et al., 2014), 35O22 (Huang et al., 2014), and 8ANC195 (Scharf et al., 2014). Kong et al. recently showed that the HIV-1 fusion peptide can also be targeted by bNAbs, specifically VRC34.01 (Kong et al., 2016). Lastly, two groups have shown that even the silent face of gp120 is a target for bNAbs with the identification of VRC-PG05 (Zhou et al., 2018) and SF12 (Schoofs et al., 2019). One epitope of note is the coreceptor binding site of Env. Early antibodies that target this site included 17b, E51, and 412d but are only effective when Env is in the CD4-bound state (Choe et al., 2003). More recently, antibodies targeting a peptide motif around the coreceptor-binding site have been described and include PGDM12 and PGDM21 (Sok et al., 2016). Although antibodies targeting the coreceptor-binding site are not as broad and potent as other classes of bNAbs, they are still useful reagents as the coreceptor-binding site is one of the most conserved regions on Env (Rizzuto et al., 1998).

With these bNAbs identified and characterized, the ideal method for employing them for HIV-1 therapy would be through a therapeutic vaccination. However, numerous limitations exist that render current vaccine approaches insufficient to elicit bNAbs. First, the bNAbs appear to arise over years of infection in a small subset of individuals living with HIV-1 (Wu et al., 2015). Second, bNAbs have long heavy chain CDR3 regions with high degrees of somatic hypermutation (reviewed in Burton and Hangartner, 2016), characteristics achievable due to chronic exposure to the virus rather than immunization. Third, properties of the virus have made immunogen design challenging. These include the low number of Env trimers on the surface of the virion (Zhu et al., 2003) as well as a protective glycan shield on Env (Wei et al., 2003). Engineering immunogens that circumvent these properties is ongoing, but currently there is no viable HIV-1 vaccine that induces bNAbs nearly 40 years after the virus was first identified. Should an effective HIV-1 therapeutic vaccine that elicits bNAbs be realized, data from clinical trials indicate that more than one active bNAb will be necessary to maintain viral suppression.

## Engineered HIV-1 Inhibitors

To address the need for targeting multiple epitopes on Env, a few groups have undertaken the engineering of bispecific antibodies and fusion proteins. Bispecific antibodies employ two techniques required for their generation. First, multiple mutations in IgG CH2 and CH3 regions of the Fc domain are introduced for a knobs-in-holes approach (Ridgway et al., 1996). These mutations link the two different IgG Fc domains at a success rate of nearly 95%. The second technique has been termed “CrossMAb” technology, where one of the two Fabs of the engineered bispecific antibody has swapped the CH1 and CL region (Schaefer et al., 2011). This technique reduces the mixing of heavy chains and light chains of the two antibodies during production of the bispecific antibody. The first demonstration of a bispecific antibody targeted the CD4-binding site and apex regions (Asokan et al., 2015). A later study used the IgG3 Fc with an open hinge that targeted the CD4-binding site and N332 V3 glycan (Bournazos et al., 2016). Both studies showed that bispecific antibodies had increased breadth compared to their individual components. One design applied to bispecific antibodies to avoid the need for a CrossMab technique is to use the full Fab of one antibody and a single-chain variable fragment (scFv) of the second antibody. A recent study by Davis-Gardner et al. applying this approach demonstrated that a bispecific antibody using the Fab from V3g bNAbs with the scFv form of CAP256-VRC26.25 had increased breadth compared to the two parent antibodies. This inhibitor maintained nearly the same potency and achieved 100% neutralization against the isolates assayed (Davis-Gardner et al., 2020). Interestingly, the most potent of bispecific antibodies comes from a design that features an HIV-1 bNAb on one arm with an anti-receptor antibody as the other arm. Huang et al. showed that their lead candidate, a bispecific antibody that targeted CD4 with one arm and used a modified 10E8 (10E8v2.0) Fab as the second arm, had 100% breadth with a mean 50% inhibitory concentration of 0.002 μg/mL against a panel of 118 HIV-1 isolates (Huang et al., 2016b).

A few groups have taken the bispecific antibody approach a step further and engineered trispecific antibodies. These antibody constructs utilize tandem scFvs fused together on one arm with a heavy and light chain from a third antibody as the other arm. Two groups showed that trispecific antibodies targeting the MPER, CD4-binding site, and either the apex or V3g had extreme coverage with great neutralization potency (Xu et al., 2017; Steinhardt et al., 2018). A third trispecific antibody recently described targeted CCR5 with an scFv and targeted the MPER and apex with the other arms of the antibody (Khan et al., 2018). Taken together, bispecific and trispecific antibodies represent single HIV-1 inhibitors with increased coverage while at least maintaining the original potency of their individual components.

Another direction of engineered inhibitors includes those that target the CD4– and coreceptor-binding sites of Env simultaneously. The first one described used a linker to fuse two domains of soluble CD4 to the scFv of 17b (Lagenaur et al., 2010). Later work has seen these kinds of inhibitors use an IgG1 Fc domain that improves the inhibitor's half-life and adds the effector functions of an antibody. One of these inhibitors came from Dimiter Dimitrov and colleagues, who used phage display to identify a stable version of CD4 domain 1 (Chen et al., 2014). This CD4 domain 1 was then fused to the N-terminus of CH1 and C-terminus of CH3 of an IgG1. Fused to the N-terminus of the CL domain is the scFv of m36.4, an antibody that targets the coreceptor-binding site. This inhibitor, termed 4Dm2m, had 100% breadth and exceptional potency against over 60 isolates it was tested against.

Our lab has taken a similar approach to developing the antibody-like HIV-1 entry inhibitor eCD4-Ig (Gardner et al., 2015). This inhibitor fuses a short, coreceptor-mimetic peptide to the C-terminus of CD4-Ig. When tested against more than 270 isolates, including HIV-2 and SIV, eCD4-Ig potently neutralizes 100% of these isolates with >99% maximum inhibition. The addition of the coreceptor-mimetic peptide also limits CD4-enhancement on CD4-negative/CCR5-positive cells. Recently we have shown that three mutations in CD4 domain 1 increase its potency by an average of 9-fold (Fetzer et al., 2018). Furthermore, *in vitro* studies showed that continuous passaging of virus in the presence of eCD4-Ig only yielded partial resistance to the inhibitor (Fellinger et al., 2019). These resistance mutations also conferred a high fitness cost to the virus in entry assays. Taken together, the broadest HIV-1 inhibitors are these fusion inhibitors that target the CD4– and coreceptor-binding sites because they target the two most conserved regions on Env.

## Adeno-Associated Virus Vectors for Delivery of HIV-1 Inhibitors

An ideal deployment of anti-HIV-1 biologics for therapy would be through constant expression to avoid lapses in maintaining therapeutic concentrations. One way to achieve this would be through the use of rAAV vectors. AAV is a small DNA virus that is widely used in numerous gene therapy applications. It was discovered as a contaminant during adenovirus preparations in 1965 (Atchison et al., 1965). AAV belongs to the *Parvoviradae* family under the genus *Dependoparvovirus*. An icosadhedral protein capsid encases its DNA genome. The wild-type AAV genome encodes three genes—*rep, cap*, and *aap* (Wang et al., 2019). The *rep* gene is responsible for the production of Rep78, Rep68, Rep52, and Rep40. These genes are necessary for replication. The cap gene encodes three subunit proteins needed for capsid assembly—VP1, VP2, and VP3. While the AAV1 capsid was the first AAV capsid identified and the AAV2 capsid has been highly characterized *in vitro*, numerous capsids have been identified and characterized for their different transduction efficiencies and tissue tropism. The third gene encoded by AAV is the *aap* gene, located in an alternate reading frame within the *cap* gene, encoding the assembly activating protein (AAP) which promotes viral assembly (Sonntag et al., 2010). As its classification implies, wild-type AAV requires a secondary infection, usually adenovirus or herpesvirus, in order to replicate. However, it is currently believed that a productive AAV infection does not cause human disease.

As a gene therapy vector, rAAV vectors are engineered to remove all parts of the AAV genome except for the two inverted terminal repeats (ITRs). The ITRs are especially important as they facilitate circularization and concatemerization in the nucleus of transduced cells (Duan et al., 1998). This process produces DNA episomes that are stable for long-term expression of the delivered transgene. The transgene encoded by rAAV is usually within the size constraints of the wild-type AAV genome (4.7 kb). However, transgenes up to 5.0 kb appear to be accommodated for vector delivery. Larger payloads can be encoded in two different rAAV vectors using splicing techniques that link the two separate portions of the delivered gene (Duan et al., 2000; Nakai et al., 2000; Sun et al., 2000).

The 2010's marked a productive decade for rAAV gene therapy. In 2012, the European Medicines Agency approved Glybera, an rAAV1-based gene therapy, which treats lipoprotein lipase deficiency. In a landmark decision, the United States FDA approved Luxturna in 2017 to treat inherited retinal disease. Luxturna is an rAAV2-based gene therapy delivering the natural form of RPE65. In 2019, Zolgensma became the second FDA-approved AAV gene therapy to treat spinal muscular atrophy in children under 2 years of age. Zolgensma is administered systemically using the AAV9 capsid to deliver the natural form of the *survival motor neuron 1* gene. A vast amount of gene therapy work has also examined treating hemophilia with rAAV vectors. Early clinical trials using rAAV2 vectors encoding Factor IX to treat hemophilia patients showed that up to 10 years after intramuscular inoculation, patients were still producing therapeutic amounts of Factor IX (Buchlis et al., 2012). This characteristic is one of the main reasons why rAAV vectors are viable for HIV-1 cure research. If long-term expression of bNAbs is critical for design of new therapies, rAAV vectors could translate into the “one-shot cure” desired by field.

While rAAV gene therapies were making headlines over the past decade, their use in HIV-1 research took off as well. All reported rAAV studies pertaining to HIV-1 research are highlighted in Table 1. Early studies were pioneered by the lab of Phil Johnson who described that rAAV2 vectors encoding the CD4-binding site antibody b12 could express the antibody for 24 weeks after intramuscular inoculation (Lewis et al., 2002). Mice given the highest dose of rAAV vectors had a range of b12 expression from 2 to 7 μg/mL and serum samples from the mice retained neutralization activity when assessed by *in vitro* neutralization assays. Johnson et al. took their research to the next step using rAAV1 vectors to deliver anti-SIV immunoadhesins and CD4-Ig to rhesus macaques (Johnson et al., 2009). In this study, four of six macaques that were expressing the anti-SIV immunoadhesins had concentrations >100 μg/mL and were protected from intravenous SIVmac316 challenges. The two animals that became infected were shown to have anti-drug antibody (ADA) responses targeting the immunoadhesin, which presumably limited the efficacy of inhibitor. Thus, this study highlights two major results. First, AAV-delivered SIV inhibitors could protect from SIV challenges, implying the potential for delivering HIV-1 bNAbs and inhibitors. Second, the host immune response is a barrier to successful utilization of rAAV vectors for therapy.

**Table 1:** List of preclinical and clinical rAAV studies using HIV-1 bNAbs and inhibitors.

**References**	**Purpose of study**	**AAV capsid**	**bNAbs**	**Virus**	**# with ADA out of total**
**Preclinical mouse studies**					
Lewis et al. (2002)	Expression	2	b12	N/A	N/A
Balazs et al. (2012)	Protection	8	VRC01, b12, 2G12, 4E10, 2F5	NL4-3	N/A
Balazs et al. (2014)	Protection	8	VRC01, b12, VRC07	JR-CSF, REJO.c	N/A
Horwitz et al. (2013)	Therapy	8	10-1074, 3BNC117	YU2	N/A
Badamchi-Zadeh et al. (2018)	Therapy	1	PGT121	JR-CSF	4/4
Van Den Berg et al. (2019)	Expression	8	CAP256-VRC26.25	N/A	7/7
**Preclinical NHP studies**					
Johnson et al. (2009)	Protection	1	4L6, 5L7, N4 immunoadhesins	SIVmac316	3/9
Gardner et al. (2015)	Protection	1	eCD4-Ig	SHIV-AD8EO	2/4
Saunders et al. (2015)	Protection	8	VRC07	SHIV-BalP4	4/4
Fuchs et al. (2015)	Protection	1	4L6, 5L7	SIVmac239	9/12
Martinez-Navio et al. (2016)	Expression, ADA response	1	4L6, 5L7, 3BNC117, 10E8, 10-1074, 1NC9, 8ANC195	N/A	24/24
Welles et al. (2018)	Protection	8	ITS01, ITS06.02	SIVsmE660	13/60
Gardner et al. (2019b)	Protection	1	3BNC117, NIH45-46, 10-1074, PGT121	SHIV-AD8EO	24/24
Martinez-Navio et al. (2019)	Therapy	1	3BNC117, 10-1074, N6, PGT128, PGT145, 35O22	SHIV-AD8EO	10/12
Gardner et al. (2019a)	Protection	1	eCD4-Ig	SIVmac239	4/4
Fuchs et al. (2019)	Expression	8, 1	4L6	N/A	12/12
**Clincal studies**					
Priddy et al. (2019)	Expression	1	PG9		10/16
Clinical trial NTC03374202	Expression	8	VRC07		2/7

*N/A, not available*.

In David Baltimore's lab, Alex Balazs and colleagues were the first to show that rAAV vectors delivering HIV-1 bNAbs could protect humanized mice from HIV-1 challenges. In Balazs et al., the authors engineered the rAAV transgene cassette to produce full-length antibodies using an F2A peptide to separate and generate both the heavy and light chains of VRC01, as well as early generations of HIV-1 neutralizing antibodies, b12, 4E10, 2F5, and 2G12 (Balazs et al., 2012). Results were extremely encouraging not only because of the observed protection from intravenous HIV-1 challenges, but also because serum concentrations of VRC01, b12, and 2G12 were >100 μg/mL. The first study using rAAV2-delivered b12 employed two different promoters to drive expression of the heavy and light chains, in contrast to this study using the F2A peptide to produce both chains and rAAV8 vectors. The F2A peptide was previously shown to drive high levels of antibody expression in mice (Fang et al., 2005), possibly indicating this design to be superior to two promoters. Balazs et al. followed up this initial study with a second study showing that VRC07W could be measured at concentrations >100 μg/mL following rAAV8 intramuscular inoculation in humanized mice (Balazs et al., 2014). These levels mediated complete protection from vaginal challenges of the transmitted-founder isolate REJO.c. These results highlighted that an rAAV-delivered bNAb could mediate protection from challenges that mimicked most HIV-1 transmission events. Overall, these two studies provided a stable foundation for AAV gene therapy in HIV-1 research. Since then, other groups have explored rAAV delivery of different bNAbs in mice, including PGT121 and CAP256.VRC26.25 (Badamchi-Zadeh et al., 2018; Van Den Berg et al., 2019).

As demonstrated in Johnson et al., the non-human primate (NHP) model presents a more difficult challenge when evaluating rAAV-delivery of HIV-1 bNAbs and inhibitors. The host immune response generated by rhesus macaques has made evaluating rAAV vectors in NHPs very difficult. The humanized mouse model has numerous benefits compared to the NHP model, one of which is that these studies utilize HIV-1 isolates. However, humanized mice do not have a fully functioning immune response like humans and NHPs do. There is also a limited amount of time for studying HIV-1 infection in mice, which limits the ability to evaluate new therapies. In contrast, the physiology of macaques more closely resembles that of humans. However, HIV-1 does not replicate in macaques. Thus, the NHP model uses SIV isolates or SIV/HIV-1 chimeras (SHIVs) for both prophylaxis and therapy studies. SIV has shown to closely mimic the disease progression to AIDS that has been observed in HIV-1 infected individuals, which makes it a good model in macaques. Yet, HIV-1 bNAbs do not bind to SIV Env so SHIVs are widely used to evaluate bNAb efficacy in NHPs. With the pros and cons acknowledged for both NHP and humanized mouse studies, there is a need to demonstrate that bNAbs are effective in NHPs to be convinced they can work in humans.

An early study by Fuchs et al. set out to evaluate the protective efficacy of the SIV antibodies 4L6 and 5L7 in rhesus macaques (Fuchs et al., 2015). Using a similar transgene cassette design as Balazs et al. with the F2A peptide to generate both the heavy and light chains, the authors used rAAV1 vectors encoding either 4L6 or 5L7 to inoculate the quadriceps muscles of rhesus macaques. Peak concentration of 4L6 ranged from 50 to 150 μg/mL while 5L7 concentrations ranged from 20 to 70 μg/mL in five of six macaques. However, the last macaque in the 5L7 group had peak and stable concentrations >200 μg/mL, a concentration that has now been achieved for over 6 years (Martinez-Navio et al., 2020). Unfortunately, antibody concentrations in most macaques began to decline due to the emergence of an ADA response against the delivered antibody. Although low-dose SIVmac239 challenges did not yield significant protection, it is of note that the macaque expressing >200 μg/mL of 5L7 was protected from all six challenges, including a high-dose intravenous challenge that has been shown to infect all historical controls. This single animal provides some encouragement that when rAAV delivery of antibody is efficient, protective efficacy will most likely be observed. However, what concentration needs to be observed in order to mediate protection is still not completely elucidated.

As Fuchs et al. described in their study, ADA was a clear hurdle to successful expression of antibodies from rAAV vectors. A similar result was observed by Saunders et al. in 2015 using rAAV8 vectors to deliver VRC07 (Saunders et al., 2015). Because VRC07 is a human-derived bNAb, the authors initially engineered VRC07 to be more “simian-like” in hopes of avoiding an immune response against the bNAb in rhesus macaques. Despite their best efforts, four of four macaques that received rAAV8 vectors encoding the simian version of VRC07 had concentrations that peaked between 2.5 and 7.7 μg/mL and then declined to concentrations below the limits of detection by 9 weeks following inoculation. The crash in VRC07 concentrations correlated with the emergence of an ADA response in all four macaques. The authors then treated a second group of six macaques with cyclosporine A (CsA) to limit the immune response of the rAAV8 inoculated animals. CsA treatment appeared to be beneficial as the mean peak concentration of VRC07 in these animals rose to 38.12 μg/mL. It was noted that one macaque generated an ADA response against VRC07 while being treated with CsA and two others generated ADA after CsA treatment was discontinued. All three of these macaques had concentrations of VRC07 decline to undetectable concentrations. However, four of six macaques were protected from a single challenge of SHIV-BalP4.

Both these NHP studies highlight the emergence of an ADA response against the expressed antibodies in rhesus macaques. Martinez-Navio and colleagues took a deeper look into their ADA responses from both their rhesus macaque study of 4L6 and 5L7 as well as rAAV1 delivery of five “rhesusized” HIV-1 bNAbs (Martinez-Navio et al., 2016). As noted in Fuchs et al., 9 of the 12 macaques that received rAAV1 vectors delivering 4L6 or 5L7 developed ADA responses. This study showed that the ADA response targeted the variable regions of the expressed antibodies. This was also observed in eight of eight macaques that received a cocktail of “rhesusized” bNAbs (bNAbs that were engineered to have the human variable regions with the rhesus macaque IgG1 constant and light chain constant regions). The two cocktails were rAAV1 vectors encoding 3BNC117, 1NC9, and 8ANC195 or 3BNC117, 10-1074, and 10E8. The authors showed that the degree of ADA responses against an individual bNAb correlated with its divergence from its closest germline precursor. The magnitude of somatic hypermutation may need to be evaluated for determining which bNAbs should be used in rAAV studies.

We have recently published a study that confirms the limitations caused by ADA responses (Gardner et al., 2019b). In our study, we took a different approach, giving four groups of three macaques a combination of rAAV1 vectors encoding either 10-1074 and 3BNC117 or NIH45-46 and PGT121. We also evaluated the role of IgG isotype as one group of each combination had a rhesus macaque IgG1 Fc domain and the other group had a rhesus macaque IgG2 Fc domain. Like Martinez-Navio et al., we observed all 12 macaques generate ADA responses against both expressed bNAbs they were treated with. In some cases, ADA responses did not completely correlate with clearance of the bNAb. In these four macaques, we observed protection from two SHIV-AD8 challenges, which indeed correlated with higher concentrations of 10-1074 or PGT121. In another NHP study, Welles et al. evaluated the delivery of anti-SIV antibodies, ITS01 and ITS06.02, using rAAV8 vectors (Welles et al., 2018). In this study, ITS01 was observed to express at higher concentrations than ITS06.02, and in only 20% of the animals that received rAAV8 vectors did macaques generate ADA responses against the expressed antibody. Stable expression was usually observed around 10 μg/mL and these concentrations were sufficient for protection from SIVsmE660 challenges. Although they have a lower level of somatic hypermutation compared to HIV-1 bNAbs, these anti-SIV antibodies were naturally produced rhesus macaque antibodies. In contrast, 4L6 and 5L7 were derived from phage display. Taken together, these studies highlight concentrations that may be protective against HIV-1 transmission but also indicate that the high amounts of somatic hypermutation of HIV-1 bNAbs may limit their protective efficacy after delivery with rAAV vectors.

In addition to studying rAAV delivered HIV-1 bNAbs, our lab has also evaluated the protective efficacy of rAAV1 expressed eCD4-Ig in rhesus macaques (Gardner et al., 2015, 2019a). In these studies, we have used the rhesus macaque CD4 domains 1 and 2 sequences with an I39N mutation that increases CD4's affinity for HIV-1 and SIV gp120 (Humes et al., 2012). We also engineered eCD4-Ig to have a rhesus macaque IgG2 Fc domain with a coreceptor-mimetic peptide fused to the C-terminus. In two different studies, two groups of four macaques each have received rAAV1 vectors encoding eCD4-Ig as well as a second vector encoding the rhesus macaque TPST2 enzyme to ensure the coreceptor-mimetic peptide gets efficiently sulfated. In our first study, macaques expressed eCD4-Ig for over 1 year with concentrations ranging from 17 to 77 μg/mL (Gardner et al., 2015). These concentrations of eCD4-Ig protected rhesus macaques from six infectious SHIV-AD8 challenges, the last challenge being 4-times higher than the dose needed to infect the last macaque in the control group. In the second study, eCD4-Ig concentrations ranged from 3 to 18 μg/mL (Gardner et al., 2019a). Although lower than the first study, these concentrations protected the treated macaques from two SIVmac239 challenges needed to infect all eight macaques in the control group. As we increased the dose of the challenges, all macaques eventually became infected with the last one getting infected at a dose 32-times greater than the dose needed to infect the macaques in the control group. Despite viral loads not being controlled after infection, we did observe eCD4-Ig-mediated pressure on the viral swarms. Three of the four macaques had noted mutations in the CD4-binding site of SIVmac239 Env, while the fourth macaque had three mutations in gp41. *In vitro* assays showed that these mutations only mediated resistance against eCD4-Ig and not complete escape, which had a clear effect on viral fitness. When looking at both studies, six of eight macaques did generate ADA responses against eCD4-Ig, however the magnitude of the responses was lower than that we observed with rAAV1-expressed HIV-1 bNAbs. In all six of these macaques, the ADA did decline over time to background levels, correlating with stable eCD4-Ig expression in these eight macaques. The lower levels of ADA against eCD4-Ig, combined with observations described in Martinez-Navio et al. and Welles et al., suggest that antibodies and inhibitors that have protein sequences closer to naturally occurring host proteins may result in long-term expression after rAAV inoculation.

The ADA issue present in NHP studies was also observed in a recent phase 1 clinical trial evaluating the delivery of PG9 from rAAV1 vectors (Priddy et al., 2019). In Priddy et al., the authors describe results from their study where participants received rAAV1 doses ranging from 4 × 10^12^ to 1.2 × 10^14^ vector genomes (vgs) inoculated intramuscularly. Importantly, no serious adverse events were noted in the study. However, PG9 concentrations were not measurable by ELISA, although the authors did note that a few participants had serum neutralizing activity against the HIV-1 NL4.3 isolate. Despite not measuring PG9 concentrations by ELISA, 10 of 16 participants did have a measurable ADA response against PG9, suggesting a low amount of PG9 was being expressed. A second clinical trial evaluating rAAV8 delivery of VRC07 (https://clinicaltrials.gov/ct2/show/NCT03374202) is currently on-going. Preliminary results were presented at the 2020 Conference on Retroviruses and Opportunistic Infections (CROI, Boston, 2020). All seven volunteers had measurable amounts of VRC07 in the plasma and two volunteers in the high-dose group (2.5 × 10^12^ vector genomes/kg) had plasma concentrations of VRC01 ranging from 1.1 to 1.2 μg/mL for more than 6 months. One encouraging note was that only two of seven volunteers had measurable ADA responses against VRC07. In the meantime, future pre-clinical studies to improve upon these two clinical trials will help determine the variables that need to be changed to yield better clinical trial results. These variables include choosing the right capsid, engineering a high-expressing AAV transgene cassette, and possibly choosing a bNAb with less somatic hypermutation than PG9 and VRC07.

## rAAV-Delivered bNAbS for the Cure

With numerous successes in demonstrating protective efficacy of rAAV-delivered bNAbs and HIV-1 inhibitors (i.e., eCD4-Ig), the question now remains whether rAAV vectors can be used for treating and curing an HIV-1 infection. Mouse studies thus far have shown what appears to be the highest concentrations of bNAbs expressed from rAAV vectors (>100 μg/mL for some bNAbs). Would these levels be sufficient for maintaining viral suppression? The NHP studies have yet to show that these concentrations can be routinely achieved in rhesus macaques. Would low concentrations of bNAbs or HIV-1 inhibitors have any impact on viral loads, let alone maintain viral suppression, or would it facilitate viral escape and render the bNAbs useless? These questions are critical for rAAV vector-mediated bNAb therapy to replace current ART.

Despite some of the current limitations observed in animal models and the PG9 clinical trial, a few studies have been encouraging. In a study published by Horwitz et al. (2013), the authors evaluated whether rAAV2-delivered 10-1074 could maintain viral suppression after ART lift (Horwitz et al., 2013). For this study, humanized mice were infected with the HIV-1 isolate YU2 and given ART to suppress infection. Five days after ART began, 10-1074 was administered via passive infusion. ART was halted after 16 days post infusion and the mice were then treated with rAAV2 vectors encoding 10-1074 12 days later. After 67 days, only one of the seven mice treated with rAAV2 vectors encoding 10-1074 had viral rebound, which was noted to have escape mutations against 10-1074. High concentrations of 10-1074 were observed, usually >100 μg/mL, which most likely played a role in viral suppression. One important note the authors made was the use of an “antibody bridge” to wash out the ART after lift and before inoculating the rAAV vectors. The need for a bridge was due to an observed decrease in antibody expression in mice on ART when inoculated with rAAV vectors. This finding may have an impact should rAAV vectors advance to clinic for treating HIV-1 infected individuals as it suggests some ART components may limit AAV transduction. The authors did address that although the results are encouraging, there are major differences between humanized mice and humans. These include the lack of viral diversity before treatment began and is highlighted by the results showing one antibody is sufficient for suppressing viremia, humanized mice are not immunologically competent, and study designs with humanized mice limit the amount of time to monitor efficacy of treatment.

While NHP studies facilitate longer-term evaluation of therapies in a host with a functional immune system, they are not capable of studying actual HIV-1 infection. SHIV isolates are routinely used for evaluating therapeutic efficacy of HIV-1 inhibitors, however, there are instances where macaques can spontaneously resolve SHIV infection without any intervention. With these limitations in mind, the Desrosiers lab published an interesting study last year looking at the therapeutic efficacy of rAAV-delivered bNAbs to suppress an on-going SHIV-AD8 infection (Martinez-Navio et al., 2019). In their first study group, four macaques received rAAV1 vectors encoding 10-1074, 3BNC117, and 10E8 at 86 weeks post infection. Unfortunately, one macaque spontaneously controlled infection immediately before rAAV1 treatment. In the remaining three macaques, one macaque (rh2438, nicknamed the “Miami monkey”) suppressed viral loads to below the limits of detection (<15 viral RNA copies/mL) for 3 years after rAAV inoculation. This macaque had high concentrations of both 10-1074 (>100 μg/mL) and 3BNC117 (>50 μg/mL) during that time frame. What makes the Miami monkey result remarkable was that these two antibodies presumably mediated viral suppression before viral escape against both antibodies could be generated. The authors also showed a drop in both viral RNA and DNA measured in PBMCs from the Miami monkey post rAAV inoculation. In the second group from this study, six macaques were treated with a combination 10-1074 and 3BNC117 or N6, 35O22, PGT128, and PGT145. All 12 animals were given rAAV8 inoculations at 36 weeks post SHIV-AD8 infection and then boosted with an rAAV1 inoculation at 60 weeks post SHIV-AD8 infection. Although none of the 12 macaques repeated the results observed in the Miami monkey, two macaques in the group that received four bNAbs demonstrated suppression, with numerous time points where viremia was below the limits of detection. In both these animals, measurable amounts of PGT128 were shown while one of the macaques also had a measurable amount of N6. Despite only reporting one Miami monkey and two additional macaques with suppressed viremia in this study, it is worth noting that the Berlin patient inspired further efforts into cure research. Thus, these results are the first steps toward demonstrating that rAAV vectors delivering bNAbs and HIV-1 inhibitors could be an alternative to ART in future years.

Our lab's current studies have been evaluating the therapeutic efficacy of rAAV-delivered eCD4-Ig in SHIV-AD8-infected rhesus macaques. In a pilot study, six macaques were treated with ART 12 weeks after infection. Two macaques received rAAV8 vectors encoding eCD4-Ig and TPST2 at 104 weeks post infection. ART was lifted 4 weeks after rAAV8 inoculation. The other four macaques received rAAV8 vectors at 56 weeks post infection and rAAV1 vectors at 70 weeks post infection. Again, ART was lifted 4 weeks after rAAV1 inoculations. In all six macaques we have observed measurable eCD4-Ig concentrations for over 2 years, ranging from 4 to 15 μg/mL. While viral rebound was observed in each macaque, we have observed subsequent viral suppression for the past year in each animal, usually with viral loads measuring <100 viral RNA copies/mL. All animals have had viral loads below the limits of detection (<15 viral RNA copies/mL) at multiple time points, and we have yet to observe viral escape from eCD4-Ig. These results are encouraging; however, they highlight the need for further vector improvement to increase eCD4-Ig concentrations. Presumably, higher eCD4-Ig concentrations could delay or suppress viral rebound after ART cessation and hopefully maintain viremia below the limits of detection in all animals over a long period of time. Additionally, because eCD4-Ig is functional against HIV-1, HIV-2, and SIV, future studies evaluating the therapeutic efficacy of rAAV-delivered eCD4-Ig against more stringent SIV isolates, like SIVmac239, would be more convincing that this therapy would translate when used in human clinical trials. What is evident in all these studies is that the next few years may be critical in advancing rAAV vectors into the clinic for preventing and treating HIV-1 infection.

## Learning From Passive Infusion Efficacy Studies

### Preclinical Studies in Humanized Mice and NHPs

Despite the limited published studies analyzing AAV-delivered HIV-1 inhibitors as a means for an HIV-1 cure, the past decade has produced several passive infusion studies that we can use as insight for the therapeutic efficacy of these inhibitors. The 2010s added to the solid groundwork from numerous studies in the late 1990s and 2000s evaluating passive infusion of first generation bNAbs (like b12 and 2G12) for SHIV protection (Mascola et al., 1999, 2000; Shibata et al., 1999; Baba et al., 2000; Parren et al., 2001; Hessell et al., 2007, 2009). The studies from the past 10 years have shown which second generation bNAbs (like VRC01, 3BNC117, 10-1074, and PGT121) have favorable properties for pharmacokinetics and are effective at suppressing an on-going infection. The first of these studies showed that individual bNAbs had little to no therapeutic efficacy against a clade B infection (YU2) in humanized mice, but a cocktail of five bNAbs yielded complete suppression of viremia, albeit, for a short period of time (Klein et al., 2012). Two studies were published a year later, where in both studies, cocktails of bNAbs were passively infused into rhesus macaques infected with SHIVs. In one study, macaques chronically infected with SHIV-SF162P3 were infused with a cocktail of three bNAbs (3BNC117, b12, and PGT121) or two bNAbs (3BNC117 and PGT121) (Barouch et al., 2013). Viremia decreased to below limits of detection in 12 of 14 animals. In the second study, rhesus macaques infected with SHIV-AD8EO were passively administered 3BNC117 and 10-1074 (Shingai et al., 2013). In 10 of 11 macaques, plasma viremia was decreased to levels below the limit of detection during treatment. However, as bNAb concentrations declined, viremia rebounded in all macaques, with resistance mutations to the bNAbs identified in the Env sequences post viral rebound. Additionally, the combination of 3BNC117 and 10-1074 in macaques acutely infected with SHIV-AD8EO could maintain viral suppression for a 56–177 days in which rebound correlated with antibody half-life. In a similar study by Hessel et al., the authors evaluated the therapeutic efficacy of PGT121 and VRC07-523 in infant rhesus macaques 24 h after being challenged with SHIV-SF162P3 (Hessell et al., 2016). As in the previous study, all animals that received the bNAb therapy were free of viremia in both the plasma and tissue measurements 6 months after treatment. A later study showed that in infant macaques, treatment with the same combination of bNAbs at 30 h post SHIV-SF162P3 challenge, but not at 48 h, was sufficient for keeping the animals from becoming infected (Shapiro et al., 2020). These studies show that bNAb therapy could be used for treating early infections if the treatment is started within a very early window of exposure.

Additional studies in non-human primates (NHPs) described that mutations in the CH3 region of the IgG1 Fc, M428L/N434S (LS), could improve the half-life and protective efficacy of bNAbs *in vivo* (Ko et al., 2014). For example, the LS mutations improved the median protective effect of 3BNC117 from 13 to 17 weeks and of 10-1074, from 12.5 to 27 weeks (Gautam et al., 2018). Of note, one macaque was protected from repeated low-dose intrarectal challenges for 37 weeks after the infusion of 10-1074 with the LS mutations. Due to these results, it is not surprising that the LS mutations have been included in evaluating the safety of bNAbs in clinical trials. LS versions VRC01 and VRC07-523 have been evaluated in the clinic and have reported half-lives of 71 and 38 days, respectively (Gaudinski et al., 2018, 2019). Having measurable quantities of bNAbs in the blood for longer periods of time would presumably lead to less frequent dosing, and ultimately, lower the cost of production.

### Clinical Studies

As more bNAbs enter phase 1 evaluation in healthy adults, a few bNAbs have already been assessed for their therapeutic efficacy in individuals living with HIV-1. In the first report from Caskey et al., 10 of 11 participants that received a 10 or 30 mg/kg dose saw plasma viremia decrease that ranged from 0.8 to 2.5 log10 viral RNA copies/mL (Caskey et al., 2015). The participant that did not respond to 3BNC117 treatment had a viral swarm resistant to 3BNC117. Although viremia rebounded in all trial participants, only some had viral swarms with changes in Env that were associated with 3BNC117 resistance. Follow-up studies based on this trial showed that 3BNC117 (and other bNAbs) could accelerate the killing of HIV-1-infected cells in humanized mice as well as improve the antibody responses against HIV-1 in participants treated with 3BNC117 (Lu et al., 2016; Schoofs et al., 2016).

Soon after, Lynch et al. reported their clinical findings for VRC01 treatment (Lynch et al., 2015). In their study, six of eight participants had a reduction of plasma viremia ranging from 1.1 to 1.8 log10 viral RNA copies/mL. Similar to that observed for 3BNC117, the two participants that did not respond to VRC01 treatment had pre-existing resistance to VRC01. Additionally, this study also showed that VRC01 treatment had no effect on cell-associated viral loads in participants treated while still on antiretroviral drug therapy. Should bNAb immunotherapy be useful for an HIV-1 cure, there will need to be evidence that bNAbs can reduce the viral reservoir (i.e., demonstrating reduction in cell-associated viral DNA measurements). A recent study showed similar results with participants acutely infected with HIV-1 (Crowell et al., 2019). These participants treated with VRC01 also had a delay to viral rebound compared to a placebo group. A third bNAb tested for immunotherapy was 10-1074 (Caskey et al., 2017). In 13 viremic individuals, 11 participants saw an average of 1.5 log10 reduction in plasma viremia after 10-1074 infusion. Overall, these three studies highlight two key points. First, bNAb immunotherapy can be effective at treating on-going infection. Second, because of the emergence of viremia after treatment which can be associated with escape, future bNAb therapies will require a cocktail of bNAbs to be most effective. To this point, a recent clinical trial evaluated the use of 3BNC117 and 10-1074 combination immunotherapy (Bar-On et al., 2018). In this study, participants that responded to treatment had an average reduction of >2 log10 viral RNA copies/mL. A few participants also had suppressed viremia for nearly 3 months after treatment and most viral isolates sequenced after therapy did not escape from the bNAb combination. These data show the promise of bNAb cocktails for therapy although future analysis will be needed to determine which bNAbs are used in combination therapy.

While these first few clinical trials showed that bNAb immunotherapy could suppress an ongoing infection, the question remains whether bNAbs have a role in eradicating virus from infected individuals. One approach has been to administer bNAb infusions while participants are on ART and then undergo an analytical treatment interruption (ATI) to monitor viral rebound. The underlying hypothesis tested in these clinical trials is that a delay in viral rebound should correlate with a decrease in viral reservoir mediated by bNAb immunotherapy. Two initial trials evaluating VRC01 in this situation indeed showed that participants treated with VRC01 while still on ART had delayed viral rebound during the ATI when compared to historical controls (Bar et al., 2016). Sequencing of the *env* gene indicated VRC01-selective pressure in the rebounding virus with most participants developing VRC01-resistant virus during rebound.

Another study evaluated 3BNC117 immunotherapy in participants that had viral loads suppressed by ART (Scheid et al., 2016). In this trial, participants that received infusions of 3BNC117 and then were taken off ART, had viral rebound ranging from 5 to 19 weeks after their final treatment. Like the VRC01 trial, these times to rebound were significantly longer when compared to historical controls. Although these results were encouraging, there was no direct analysis to definitively show that 3BNC117 immunotherapy decreased the latent reservoir. However, it was shown that rebound virus during ATI in participants treated with 3BNC117 was not the predominant species observed in the latent reservoir prior to ATI (Cohen et al., 2018). Additionally, although all participants were screened to be sensitive to 3BNC117 immunotherapy, resistance mutations were identified in the latent reservoir before treatment was initiated. Thus, this study is another example for the need of bNAb combinations even when participants are pre-screened for sensitivity to one bNAb. Mendozza et al. have reported successful results when using the combination bNAb immunotherapy using 3BNC117 and 10-1074 (Mendoza et al., 2018). Participants that received this therapy had suppressed viremia for a median of 21 weeks after ATI was initiated. Viral rebound was observed in all but two participants, and this usually correlated with the clearance of 3BNC117, meaning the participants were effectively on monotherapy at the time of rebound.

Overall, these results are very encouraging in that, at the very least, bNAb combinations can suppress viremia without ART for relatively long periods of time and could be a future alternative to current ART. However, for passive infusion of bNAbs to work as an HIV-1 therapy, individuals will continuously need to receive these infusions, multiple times a year and thus, a requirement for constant production of bNAbs would be needed. Considering the average American male and female are roughly 90 and 77 kg, respectively, more than two grams of bNAbs would be needed per treatment. The underlying question is whether this type of treatment is feasible on a global scale.

## Current Limitations When Using rAAV Vectors

**Figure 1:**
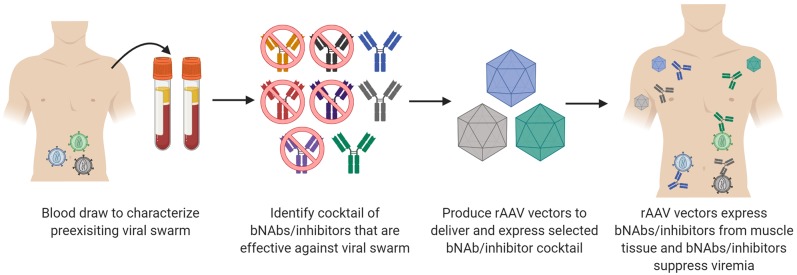
Process for selecting a bNAb/inhibitor cocktail for delivery by rAAV vectors. Similar to ART, it is highly unlikely that a single bNAb or inhibitor cocktail will be effective for every individual living with HIV-1. Therefore, cocktails will most likely be tailored to the viral swarm present for each individual or endemic region. Initial screening will need to be conducted on the preexisting viral swarm to identify which bNAbs or inhibitors have activity against that swarm. rAAV vectors that encode each of the individual components of the cocktail will need to be produced. Delivery of the rAAV vectors will then yield a cocktail of inhibitors made by individual that target the HIV-1 swarm and suppress infection.

In order to pursue clinical trials evaluating the therapeutic efficacy of bNAbs delivered by rAAV vectors, three areas of research will be critical to success. The first is identifying which bNAbs or HIV-1 inhibitors should be used in a cocktail of antibodies. No single bNAb described to date can neutralize 100% of HIV-1 isolates tested. Thus, it is a forgone conclusion that multiple bNAbs will be necessary to treat an ongoing HIV-1 infection. The clinical trials have especially shown this in that individual bNAb therapy results in viral rebound in both ART-treated participants and participants undergoing ATI. However, the clinical data that shows long-term suppression after ATI when combining 3BNC117 and 10-1074 underscores the value of bNAb combinations for therapy (Mendoza et al., 2018). Mathematical modeling has been used to identify the best bNAb combinations for specific areas with HIV-1 infected populations (Wagh et al., 2016, 2018). Combinations of bNAbs and bispecific antibodies may be even more effective than bNAbs alone. However, bispecific antibodies provide a challenge for rAAV vectors in that both arms of the antibody cannot fit into a single rAAV transgene cassette. eCD4-Ig may be a useful reagent to combine with bNAbs as current studies show that it neutralizes all HIV-1 and HIV-2 isolates tested. Additionally, viral resistance mutations against eCD4-Ig come with a fitness cost to the virus. Binding studies have shown that eCD4-Ig may pair well with V3g bNAbs (Davis-Gardner et al., 2017). eCD4-Ig also has the benefit at enhancing the ADCC activity of non-neutralizing antibodies that are elicited during an HIV-1 infection (Davis-Gardner et al., 2017). What still needs to be shown is that high concentrations of eCD4-Ig can be expressed routinely from rAAV vectors, and that eCD4-Ig is safe and well-tolerated after passive infusion in phase I clinical trials. As shown in Figure 1, the process for identifying the correct cocktail will likely be dependent upon the viral swarm present in the individual living with HIV-1. Viral swarms will need to be characterized for each individual to determine an active cocktail of bNAbs and inhibitors to use on that swarm. Is this necessary? Most likely. Is it practical for the millions of people living with HIV-1? Possibly not as it would require that manufacturing and production of numerous rAAV vectors encoding many different bNAbs or inhibitors.

A second area of research that requires more analysis is the ability for bNAbs, bispecific antibodies, and HIV-1 inhibitors to reduce the latent viral reservoir. Both clinical and pre-clinical bNAb therapy studies have yet to definitively show an increase in rate of reduction of the latent viral reservoir. The latent reservoir is a tough target as it remains hidden in numerous tissue compartments like lymph nodes and gut-associated lymphoid tissues. Can bNAbs and HIV-1 inhibitors access these tissue compartments to kill infected cells and would they reach these tissue compartments when expressed from AAV vectors? Will we need latency reversal agents (LRAs) to stimulate virus production to generate infected cells for bNAbs to target? Are the current LRAs good enough to be combined with bNAb immunotherapy to reduce the viral reservoir? A few studies have investigated these questions. Advancements in visualizing exactly where these antibodies go once delivered into a host include fluorescent imaging of tagged-bNAbs (Schneider et al., 2017) and using PET scans in animal models. Additionally, there is a clinical trial using radiolabeled VRC01 that is in the recruiting phase to use PET-MR imaging that will give insight into bNAb distribution in humans (https://clinicaltrials.gov/ct2/show/NCT03729752). These studies will then lead to down-stream questions about whether bNAb and HIV-1 inhibitors expressed from AAV vectors get distributed to the same areas of the host. Pertaining to stimulating viral production in infected cells, the TLR7 agonist GS-9620 induced viremia in SIVmac251-infected rhesus macaques that had viremia suppressed by ART (Lim et al., 2018). In another study, macaques infected with SHIV-SF162P3 and with viremia suppressed on ART were treated with GS-9620 and PGT121 immunotherapy (Borducchi et al., 2018). This group of treated animals had a delay in viral rebound and lower set point viremia in 6 of the 11 macaques that had rebound after ART lift. Of note, in this study GS-9620 did not induce transient viremia. Thus, other reagents may be more effective to reverse latency and induce viral replication. SMAC-mimetic inhibitors that stimulate the non-canonical NF-kB pathway have shown promise as future LRAs to evaluate with bNAbs in NHP studies (Pache et al., 2015; Nixon et al., 2020). Additionally, an IL-15 superagonist (N-803) coupled with CD8+ T cell depletion could provide another avenue to explore (Mcbrien et al., 2020). Combining these LRAs with rAAV-delivered bNAbs or HIV-1 inhibitors could provide valuable insights for HIV-1 cure research. Dosing LRAs in the presence of persistent expression of bNAbs or HIV-1 inhibitors could provide the field with the data necessary for determining whether the shock-and-kill approach reduces the viral reservoir.

**Figure 2:**
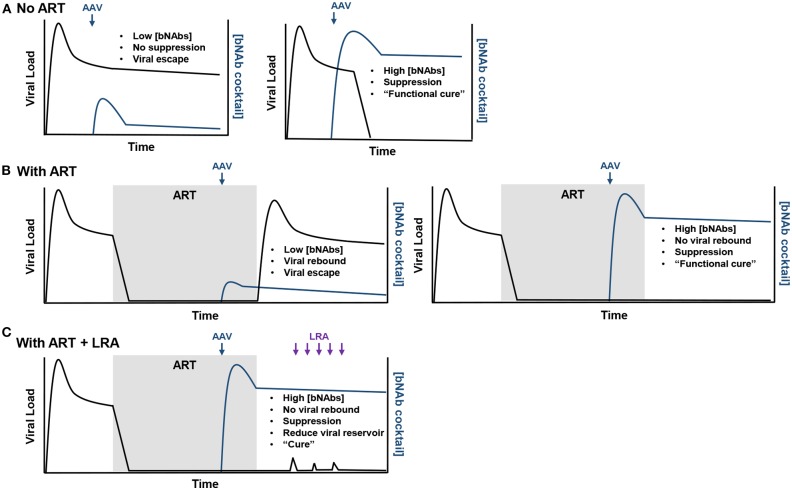
Possible outcomes from rAAV-mediated bNAb therapy. **(A)** In the scenario where rAAV-delivered bNAbs (blue arrow) are used to treat chronic infection, if the bNAb concentrations do not reach therapeutic levels, no viral suppression will be observed and viral escape from the bNAbs will be likely (left). However, therapeutic concentrations may suppress viremia and provide a functional cure for the individual (right). **(B)** In individuals treated with ART, low concentrations of rAAV-expressed bNAbs will likely result in viral rebound (left). High concentrations of bNAbs will maintain viral suppression after ART lift and provide a functional cure (right). **(C)** Should therapeutic concentrations maintain viral suppression after ART lift, LRAs (purple arrows) may be administered to stimulate replication (viral load blips) in latently infected cells and provide a target for bNAbs. If this approach is successful, a measurable decrease in the viral reservoir should be observed and eventually lead the individual to being cured of the HIV-1 infection.

Potential outcomes of rAAV-delivered bNAbs combined with and without LRAs are highlighted in Figure 2. First, should individuals living with HIV-1 not treated with ART receive subtherapeutic concentrations of bNAbs, viremia will remain stable and viral escape mutations are likely to occur (Figure 2A). However, high concentrations of bNAbs, like that observed in the Miami monkey, may be able to suppress infection and result in a functional cure. Second, expression after rAAV inoculation that is again below the therapeutic threshold in individuals with suppressed viremia from ART will likely result in viral rebound and escape during ATI (Figure 2B). Yet, should high concentrations of bNAbs be observed, there is a possibility of no viral rebound during ATI, such as that observed in the clinical trial that used 3BNC117 and 10-1074 immunotherapy. Lastly, in the best-case scenario where a functional cure is observed using rAAV-delivered bNAbs, LRA administration may help stimulate viral replication that could ultimately reduce the viral reservoir (Figure 2C). The only way to cure individuals living with HIV-1 is through this scenario where viral suppression is maintained and the viral reservoir is reduced by bNAbs.

A third area of research that needs to be resolved for rAAV vectors to work is the immunogenicity issues. As highlighted in the NHP studies and confirmed in the PG9 clinical trial, ADA responses against the expressed bNAb may limit measurable concentrations and functionality of the bNAb. Interestingly, ADA against eCD4-Ig appears to decline over time. Is it possible to engineer bNAbs to have such a quality or do we need to identify the best bNAbs with least amount of somatic hypermutation to use for rAAV delivery? Would these be the bNAbs that work best in combination for treating HIV-1 infection? Additionally, the rAAV capsid presents its own immunogenicity issues. Delivery of rAAV vectors will likely cause T cell and antibody responses against the capsid, limiting the number of times a capsid can be used to just one dose (Ertl and High, 2016). T cell responses also play a role in removing transduced cells, thus lowering the total concentration of expressed transgene. Mouse studies have shown that different AAV capsids do have different immunogenicity properties. For example, the AAV1 capsid appears to be more immunogenic than the AAV8 capsid as it stimulates greater T cell responses (Lu and Song, 2009). Generation of regulatory T cells (Tregs) has also been correlated with lower immune responses against rAAV delivered transgenes (Cao et al., 2007). Inducing tolerance to rAAV transgene is thus an interesting approach to promote persistence of transgene expression. One way to promote tolerance has been to target the liver with rAAV vectors to produce more Tregs. In a recent study, intravenous administration of rAAV8 vectors encoding 4L6 followed by an intramuscular rAAV1 vector inoculation 14 weeks later, resulted in robust 4L6 concentrations in three of three rhesus macaques (Fuchs et al., 2019). Thus, the liver expression of rAAV transgenes may provide a means of tolerance. However, liver transduction may be complicated by another immunogenicity factor. Pre-existing neutralizing antibodies against specific AAV capsids limit the ability to use specific capsids for transduction. Studies evaluating the seropositive status of humans against AAV capsids have shown that a high prevalence of the human population already has neutralizing antibodies against at least one AAV capsid (Halbert et al., 2006; Calcedo et al., 2009; Boutin et al., 2010; Hüser et al., 2017). It has also been shown that AAV neutralizing antibodies do not have as much of an effect when inoculations are administered intramuscularly (Greig et al., 2016), which makes skeletal muscle tissue an easier target compared to the liver. Another approach could be through the use of synthetic capsids (Büning and Srivastava, 2019; Pekrun et al., 2019). Both rational design and directed evolution studies are being employed to generate synthetic capsids that improve AAV transduction to specific tissues and avoid the pre-existing neutralizing antibody responses. Another issue is that the AAV genome itself is immunogenic as it is a target of the TLR9 pathway (Zhu et al., 2009). Genome engineering studies, such as CpG depletion (Faust et al., 2013), have been effective at lowering the TLR9 response but studies in NHPs are warranted. Overall, overcoming the numerous host immune responses against AAV will be critical for rAAV vectors to be utilized in the clinic, not only for delivering HIV-1 bNAbs and inhibitors, but for other gene therapies as well.

Lastly, the safety of AAV gene therapy vectors will need to be addressed. Ideally, systems would be in place that can regulate transgene expression in case adverse events are observed after infusion. A few studies have delved into regulating transgene expression in AAV systems. Some studies investigating transgene regulation can induce expression using small molecules (Yen et al., 2004; Mou et al., 2018; Zhong et al., 2020). However, in a situation where AAV vectors are used for delivery anti-HIV-1 biologics for a cure, ideally the vector would constitutively express the inhibitor and contain elements for a kill-switch. A functional kill-switch for AAV vectors has yet to be realized, but it can be imagined that Cre/LoxP or CRISPR/Cas systems might solve the issue in the coming years. Overcoming the safety concerns as well as the other hurdles that were noted would improve a gene therapy system that already has a great foundation. The promise that AAV gene therapy provides for treating numerous diseases supports future studies to solve these issues.

## Summary

A true cure that eliminates the HIV-1 reservoir is a formidable goal, yet one that is worth pursuing. As noted in this review, bNAbs and HIV-1 inhibitors may play a role as part of an HIV-1 cure, but questions persist whether these drugs can ultimately impact the rate of decay of the reservoir. The Miami Monkey provides the field with evidence that rAAV vectors could be part of a “one-shot cure” in that these vectors could provide long-term expression of bNAbs and inhibitors and at therapeutic concentrations, suppress an HIV-1 infection in place of daily ART. Thus, a pathway to curing HIV-1 using bNAbs or inhibitors is a functional cure without adherence to ART. Further studies are warranted combining rAAV-delivered bNAbs and HIV-1 inhibitors with LRAs to determine whether the shock-and-kill approach is viable with these reagents. Overall, the future research in the HIV-1 cure field remains promising as we enter a new decade with an arsenal of new and effective tools at our disposable.

## Author Contributions

MG composed the manuscript and figures.

## Conflict of Interest

MG is a co-founder and shareholder of Emmune, Inc., a start-up company dedicated to the development of eCD4-Ig and AAV-delivered eCD4-Ig for clinical trials.
